# Diabetes and Metabolic Disorders: Their Impact on Cardiovascular Events in Liver Transplant Patients

**DOI:** 10.1155/2023/2199193

**Published:** 2023-06-23

**Authors:** Simone Di Cola, Giulia Cusi, Lucia Lapenna, Jakub Gazda, Stefano Fonte, Marco Mattana, Gianluca Mennini, Patrizio Pasqualetti, Manuela Merli

**Affiliations:** ^1^Department of Translational and Precision Medicine, Sapienza University of Rome, Viale Dell'Università 37, Rome 00185, Italy; ^2^2nd Department of Internal Medicine, Pavol Jozef Safarik University and Louis Pasteur University Hospital, Trieda SNP 1, Kosice 040 11, Slovakia; ^3^Department of Hepato-Biliopancreatic and Transplant Surgery, Sapienza University of Rome, Viale Del Policlinico 155, Rome 00161, Italy; ^4^Department of Public Health and Infectious Diseases, Faculty of Pharmacy and Medicine, Sapienza University of Rome, Piazzale Aldo Moro 5, Rome 00185, Italy

## Abstract

Cardiovascular diseases are currently one of the most important causes of morbidity and mortality in liver transplant patients over the long term. Therefore, evaluating prognostic factors for cardiovascular events (CVEs) in this population is essential for taking preventive measures. The aim of this study was to identify the impact of diabetes and other metabolic disorders on CVEs in liver transplant patients. Three hundred fifty-six liver transplant recipients who survived at least 6 months after surgery were enrolled. Patients were followed for a median time of 118 months (12–250 months). All cardiovascular events were carefully recorded and detailed in the patients' charts. Demographic data, diabetes, hypertension, dyslipidemia, weight changes, and a diagnosis of metabolic syndrome both before and after transplantation were noted to assess their possible relationship with CVE. The presence of a diagnosis of metabolic-associated fatty liver disease (MAFLD) was also evaluated. Immunosuppressive therapy was included in the analysis. Diabetes mellitus (DM), especially when present before transplantation, was strongly associated with CVEs (hazard risk HR 3.10; 95% confidence interval CI: 1.60–6.03). Metabolic syndrome was found to be associated with CVEs in univariate analysis (HR 3.24; 95% CI: 1.36–7.8), while pretransplantation and de novo MAFLD were not. Immunosuppressive therapy had no influence on predisposing transplanted patients to CVEs during follow-up. Further prospective studies may be useful in investigating the risk factors for CVEs after liver transplantation and improving the long-term survival of transplant patients.

## 1. Introduction

Liver transplantation (LT) has become a well-established treatment for end-stage liver disease due to advancements in surgical techniques and immunosuppressive therapies. As a result, the survival rate for liver transplant patients has significantly improved over the years. In Europe, survival rates are 84% and 72% at 1 and 5 years after transplantation, respectively [1].

While the short-term survival rates of liver transplant patients continue to improve, various long-term complications have drawn the attention of clinicians. These complications might be related to liver disease recidivism, posttransplant metabolic changes, and chronic immunosuppressive therapy [2, 3].

Furthermore, the progressive increase in age of the surviving population is associated with increased cardiovascular morbidity and mortality, with the cardiovascular risk being even higher in transplant recipients than in the age- and sex-matched control population [4].

This increased risk has been attributed to various factors, including those intrinsic to the patient and those related to transplantation, such as the requirement for long-term immunosuppressive therapy to prevent organ rejection [5, 6]. Metabolic alterations, such as diabetes mellitus, arterial hypertension, dyslipidemia, obesity, and hepatic steatosis may already be present before the transplant; furthermore, the majority of liver transplant recipients develop a metabolic syndrome after transplantation, which can contribute to an increased risk of cardiovascular events in the long term [7–10].

Diabetes is a significant risk factor for cardiovascular events (CVEs) in liver transplant patients [11–14], and some studies have suggested that de novo diabetes may be an even more relevant predictor of CVEs after transplantation [15]. Other metabolic conditions besides diabetes are also relevant. De novo hypertension is a common complication, affecting 50–75% of patients [8, 9, 16], and 20–60% of recipients report hyperlipidemia [17, 18]. The kind of immunosuppressant (such as the calcineurin inhibitor and the mTOR-Mammalian target of rapamycin inhibitors) may also contribute to these alterations [5, 19]. While weight gain after surgery is often desirable to correct malnutrition, continued weight gain can lead to overweight status, affecting approximately 50+% of transplant recipients three years after surgery [20], and the psychological conditions that occur in patients who undergo transplantation can play a fundamental role in this setting [17]. Studies have shown that obesity has a significant impact on long-term morbidity and mortality after liver transplantation in Europe. Additionally, the prevalence of metabolic syndrome after transplantation is estimated to be between 30 and 50% [8], which can increase cardiovascular risk and cause the development of hepatic steatosis in the new graft [21].

Recently, a new definition called MAFLD (metabolism-associated fatty liver disease) has been coined [22]. Indeed, MAFLD is diagnosed when there is documented fatty liver disease associated with overweight/obesity and/or the presence of type 2 diabetes mellitus or evidence of at least two of the seven metabolic dysregulations. MAFLD has been proposed as a contributing predictive factor of cardiovascular risk in the general population, with more capacity to identify people at a higher risk of CVEs and all-cause mortality as compared to the definition of NAFLD [23, 24]. The prevalence and relevance of MAFLD in the liver transplant setting are still unknown.

The aim of this study was to evaluate the association of metabolic alterations, including MAFLD, and immunosuppressive treatment with the incidence of CVEs in liver transplant patients over time.

## 2. Materials and Methods

### 2.1. Patients

This retrospective study aimed to analyze the demographic and clinical data of patients who underwent elective liver transplantation at the University Hospital Policlinico Umberto 1 between January 2000 and December 2020. The Ethics Committee of the Sapienza University of Rome approved this study (EC Prot. 0560/2022, 06/07/22). Before transplantation, all patients from our center undergo a rigorous cardiovascular assessment, which includes electrocardiography, echocardiography, and intracardiac pressure measurements if pulmonary hypertension is suspected. In some cases, cardio-CT or coronarography is used to exclude cardiac ischemia.

To be eligible for this study, patients had to have a follow-up of at least 12 months at the center. After excluding 33 patients with less than 12 months of follow-up and 27 patients who continued their follow-up at other regional tertiary centers, a total of 356 patients were enrolled in the study.

### 2.2. Definitions

A composite variable “cardiovascular event” was created for the purpose of the study, and it included transient ischemic attack (TIA), ischemic and hemorrhagic stroke, cardiac decompensation, cardiac arrest, acute myocardial infarction (MI), symptomatic non-MI ischemic heart disease, and ischemia-based peripheral vasculopathy.

### 2.3. Data Collection

The data analyzed were extracted anonymously through a thorough review of patients' charts. The data collected before transplantation included age, sex, reason for transplantation, body mass index (BMI), and presence of metabolic comorbidities such as diabetes (transient posttransplant diabetes lasting less than 6 months during steroid treatment was not considered) [25], arterial hypertension, dyslipidemia, obesity, metabolic syndrome, and liver steatosis. Data were also collected at the last follow-up postliver transplantation, and they included changes in body weight and BMI, occurrence of de novo diabetes, arterial hypertension [26], dyslipidemia [27], overweight or obesity [28], complete metabolic syndrome, or liver steatosis. The definitions of these conditions are provided in the guidelines cited. Details about immunosuppressive therapy were extracted, and the presence of MAFLD [22] was investigated in all patients before and after transplantation if information was available. Additionally, if a patient suffered a major cardiovascular event, their age, time from transplantation, and clinical outcome were recorded. The date and cause of death were also extracted.

### 2.4. Statistical Analysis

Clinical and demographic characteristics were reported using the mean and standard deviation or median and interquartile range (IQR) based on the distribution's normality. Categorical variables were expressed as absolute counts and percentages.

We did not perform prior sample size calculations because of the retrospective nature of the study. However, all eligible data available were considered to maximize the power and generalizability of the results.

Univariable and multivariable models were analyzed using complete case analysis. The only potentially relevant predictors with missing values were metabolic syndrome (34 missing values) and MAFLD (100 missing values). Multiple imputation for missing data was performed for the metabolic syndrome by replacing missing values with predicted group membership according to a multivariable logistic model. For MAFLD, no imputation was conducted due to the large number of missing values (28%) compared to the entire sample.

Variables with *p* values <0.10 in the simple model were included in the multivariable model using a change-in-effect criterion. Absolute risks were calculated as the number of events that occurred in a group divided by the number of people in that group.

To compare the risk of CVEs among nondiabetic, pretransplant DM patients and *de novo* DM patients, traditional proportional-hazard Cox regression and Fine-gray competing risk models were utilized, with CVEs as the primary outcome and CVE-unrelated mortality as a competing event. In the multivariable model, we adjusted for demographic characteristics that may be associated with CVEs or death, such as age at LT, sex, etiology of liver disease, hypertension, hyperlipidemia, MAFLD, metabolic syndrome, and treatment.

The significance of differences in continuous variables between groups was tested using Fisher (*F*) or Mann–Whitney tests with Bonferroni corrections.

## 3. Results

### 3.1. General Characteristics of the Entire Cohort

A total of 356 patients were included in the study, and they comprised 277 (77.8%) men and 79 (22.2%) women.

The median age at the time of transplantation was 56 years in the whole population, with no differences between males and females.

The median duration of follow-up was 118.5 months, with a range of 12 to 250 months. The most common underlying chronic liver diseases were alcohol-related liver disease (*n* = 107, 30.1%) and HCV and/or HBV infection (*n* = 196, 55.1%), followed by autoimmune and/or cholestatic liver disease (*n* = 20, 5.6%) and nonalcoholic hepatitis (*n* = 20, 5.6%), with other diseases accounting for 56 cases (15.7%). Hepatocellular carcinoma (HCC) was concurrently diagnosed in 173 patients (45.8%) (Table 1).

Regarding the immunosuppressive maintenance therapy, 192 (53.9%) patients were treated with monotherapy of calcineurin inhibitors, while 47 (13.2%) were prescribed everolimus or sirolimus monotherapy. The remaining 117 patients (32.9%) received various combined therapies. Of these 60 patients (16.8% of the entire cohort) treated with tacrolimus and mycophenolate mofetil, 28 patients (7.8% of the entire cohort) were treated with cyclosporine and mycophenolate mofetil, 1 patient (0.3% of the entire cohort) was treated with sirolimus and mycophenolate mofetil, 4 patients (1.2% of the entire cohort) were treated with azathioprine and tacrolimus, and 24 patients (6.7% of the entire cohort) were treated with tacrolimus and everolimus.

During the follow-up period, a total of 78 (21.9%) patients died. Among them, 21 (26.9%) patients died due to the recurrence of liver disease (such as the recurrence of viral cirrhosis, cholestatic liver disease, and alcohol relapse), 18 (23.1%) died due to the diagnosis of malignancies (of these, 5 patients died for HCC recurrence, the remaining 13 patients from de novo malignancies), 11 (14.1%) died due to infections, and 5 (6.4%) died due to cardiovascular disease.

The median BMI value at the time of transplantation was 25 kg/m^2^ (IQR 22.6–28.1), whereas the median value after transplantation was 25.9 kg/m^2^ (IQR 23.2–28.7). In particular, 15.8% of patients were obese (31.9 kg/m^2^—IQR 30.8–34 kg/m^2^), and 34.5% were overweight before transplantation (26.8 kg/m^2^—IQR 25.7–28.1 kg/m^2^). After transplantation, the percentage of obese patients increased to 18.0% (32.3 kg/m^2^—IQR 31.2–34.2 kg/m^2^), while the proportion of overweight patients increased to 40.9% (27.3 kg/m^2^—IQR 26.0–28.4 kg/m^2^). Overall, after transplantation, the majority of patients (58.9%) had a BMI >25 kg/m^2^. BMI at the time of transplantation was significantly related with the origin of liver disease (*F* (4,276) = 9.584, *p* < 0.001) since patients with alcohol-related cirrhosis (27.0; 95% CI: 25.8–28.1) and with nonalcoholic steatohepatitis (NASH) (29.6; 95% CI: 27.3–31.9) had a higher BMI than patients with viral (25.2; 95% CI: 24.6–25.8) or autoimmune hepatitis (22.9; 95% CI: 20.9–25.0). This significant difference persisted even during the posttransplant follow-up period. The modifications of BMI after LT were not significantly different depending on the origin of liver disease (*F* (5,307) = 0.46; *p*=0.807).

Prior to transplantation, 93 (26.1%) out of 356 patients had been diagnosed with diabetes. Among them, 8 patients exhibited regression of diabetes following the surgery. Seventy-four (20.8%) patients developed de novo diabetes after transplantation, resulting in a total of 159 (44.7%) patients with diabetes after transplantation. We investigated whether patients who developed de novo diabetes had different demographic and clinical characteristics compared with patients with pretransplant diabetes and nondiabetic patients. However, no significant differences were found (Table 1).

Metabolic syndrome was present in 17 patients (5.3%) prior to liver transplantation. However, its prevalence increased from 5.3% to 10.6%, as 34 patients developed this syndrome following liver transplantation.

Moreover, the percentage of patients developing de novo metabolic alterations after transplantation is noteworthy: 20.8% of patients became diabetic, 40.2% of subjects developed arterial hypertension, and 43% of patients showed dyslipidemia. A smaller percentage of patients also developed either MAFLD or complete metabolic syndrome (as shown in Table 2).

### 3.2. Cardiovascular Events

After transplantation, 63 (17.7%) cases of cardiovascular events (CVEs) were documented. The characteristics of the two cohorts of patients (with CVE and without CVE) are reported in Table 1.

During the follow-up, as reported in Table 3, myocardial infarction, stroke, and a diagnosis of peripheral vasculopathy were the more frequent events.

The occurrence of CVEs took place on average 71 months after transplantation, with a range of 12 to 228 months. Of the entire cohort, 5 (7.9%) patients died due to CVE-related complications.

### 3.3. Factors Associated with Cardiovascular Events

Simple and multiple Cox regression analyses and the fine-gray competing risk regression analysis are reported in Table 4. The following variables were found to be associated with posttransplant CVEs in simple Cox regression analyses: male sex, higher age, alcohol-related liver disease, higher BMI, pretransplant diabetes mellitus, pretransplant arterial hypertension, and de novo arterial hypertension. In the multiple Cox regression analysis, female sex, age, and pretransplant DM remained associated with posttransplant CVEs. In the multiple fine-gray competing risk regression analysis, female sex, age, and pretransplant DM were all found to be associated with posttransplant CVE (the “diabetes” variable was introduced to the multivariable analysis because of significant HR in the univariable analysis).

A diagnosis of MAFLD, neither before nor after liver transplantation, was not associated with CVE, similar to viral hepatitis and the type of immunosuppressive therapy. On the other hand, a diagnosis of metabolic syndrome before liver transplantation was significantly associated with CVEs (Table 4).

### 3.4. Impact of Pretransplant vs. De Novo DM on CVE

**Figure 1:**
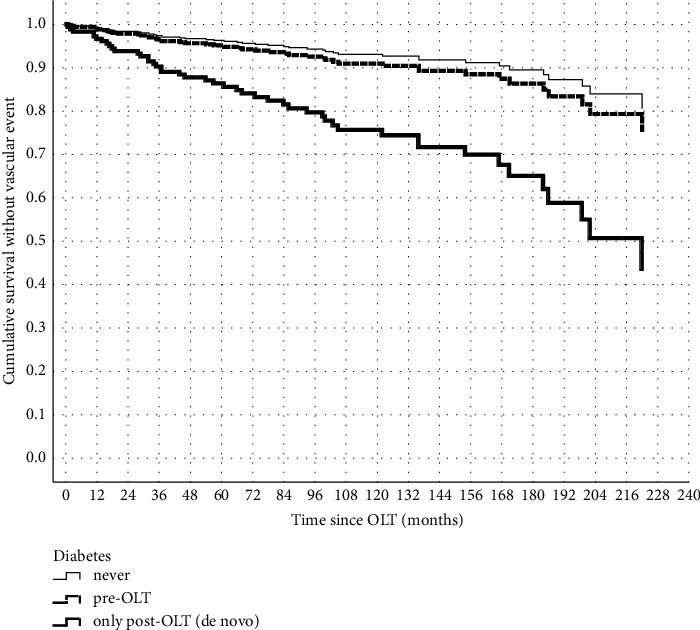
Kaplan–Meier curve of CVE‐free survival in nondiabetic patients, pretransplant DM patients, and de novo DM patients.

Patients with pretransplant DM had the highest incidence of CVE (31.1%), while those with de novo DM had a lower incidence at 16.1%, and nondiabetic patients had the lowest incidence at 10%. At 5 years postliver transplantation, 20% of subjects with pretransplant DM had experienced a CVE, whereas only 5% of those with de novo DM had experienced one. After 10 years, the respective percentages were 30% in patients with pretransplant diabetes mellitus (DM) and 10% in patients with de novo DM (Figure 1).

## 4. Discussion

Over time, success and survival rates following liver transplantation have risen significantly, resulting in an increasing prevalence of long-term complications. Among these, metabolic complications are common as a consequence of multiple etiologic factors. Cardiovascular events are one of the leading causes of morbidity and mortality in the Western world and are closely linked to metabolic comorbidities.

Our retrospective study aims to explore how metabolic features can be modified in a population of transplant patients and how they may impact on CVEs after transplantation. For this purpose, we analyzed multiple parameters before and after liver transplantation.

In the case of our cohort, viruses and alcohol were the most frequent causes of liver cirrhosis. A moderate percentage of these patients had already been diagnosed with arterial hypertension, diabetes, and dyslipidemia during the pretransplant period (20.5%, 26.1%, and 3.9%, respectively). The recent increase in metabolic cirrhosis as a condition leading to liver transplantation may increase these numbers further in more recent cohorts [29].

During a follow-up period of an average of 10 years after LT, the prevalence of metabolic alterations increased steeply (Table 2), as also previously reported [4].

A consistent number of subjects also presented with issues of overweight and obesity. Weight gain after transplantation may occur due to the modification of dietary habits, increased appetite, and the effect of immunosuppressive drugs (especially as corticosteroids increase hunger and fluid retention) [30]. In addition, in the population transplanted because of alcohol-related liver disease, a transfer of the craving phenomenon from alcohol to food frequently occurs, with increased calorie intake and weight gain [31]. This trend was also present in our cohort.

During the study period, 63 CVEs were observed. The most frequently occurring of these were stroke and myocardial infarction.

CVEs were associated with sex, age, arterial hypertension, dyslipidemia, diabetes, and the presence of the metabolic syndrome. Our multivariate analysis selected the male sex, older age when transplanted, and patients presenting with diabetes and dyslipidemia. The results of other studies are consistent with ours, thus confirming how important it is to focus on these factors during patient follow-up [13, 15, 32]. A review of metabolic complications in liver transplant patients and their management was recently published [33]. The cumulative incidence of cardiovascular disease in the 8 years following transplantation yielded a percentage of 30%. The following factors were identified as risk factors for CVEs: diabetes, dyslipidemia, arterial hypertension, obesity, cigarette smoking, and renal insufficiency. Pretransplant diabetes was identified in 33–66% of patients (26% of the cases included in our cohort) and severely affects the prognosis of the transplanted patient in terms of mortality, CVE, and infection rates. Dyslipidemia was present after liver transplantation in 45–71% of cases (compared with approximately 46% for our cohort) and responded poorly to lifestyle modifications. In our study, approximately 60% of the patients presented with arterial hypertension present after transplantation, a value similar to that of 70% reported in the review.

One of the most frequently acknowledged CVE risk factors in liver transplant patients is diabetes. Two broad-scale studies reported in the literature have, however, provided conflicting results on this particular point [15, 34].

Our single-center study found results similar to those of Kuo et al. [34]. Indeed, we found that a diagnosis of diabetes before transplantation was associated with an increased risk of CVEs (HR 2.90; 95% CI 1.62–5.19), while this association was not significant in patients who experienced de novo DM (HR 1.37; 95% CI 0.69–2.72) (Table 4).

The duration of DM is included in the calculation of CV risk [35], and the risk is reported to be high if the duration is more than 10 years and very high if it is more than 20 years. In our study, the length of the diagnosis of DM before liver transplantation was not available; however, we can assume that the exposure to higher glucose levels lasted for a longer period in these patients in comparison with patients with de novo DM.

In patients with cirrhosis of nonmetabolic origin, the coexistence of low cholesterol levels (following reduced liver synthesis) and reduced mean arterial pressure (as a consequence of vasodilatation) may decrease the rate of cardiovascular complications but the interaction between diabetes and liver disease is likely to be more complex. Some longitudinal studies reported that the low CV complications in cirrhotic patients with diabetes was explained by the higher mortality, mostly related to cirrhosis-related complications, in these patients [36].

A second recognized risk factor for CVE is the presence of metabolic syndrome (MS). The diagnosis of MS should therefore be sought and promptly addressed with multidisciplinary approaches. However, the diagnosis of MS can be complex and fluctuating after liver transplantation, influenced by collateral effects due to immunosuppressive therapies and the trend to increase body weight.

In this regard, the concept of MAFLD has also emerged, as it has broader diagnostic criteria and allows for identifying a segment of the population at risk that is not included in the definition of MS. Unfortunately, only a subgroup of patients in our cohort had complete data for the assessment of MAFLD. Therefore, the number of patients with MAFLD could have been underestimated. Despite this bias, in contrast to the normal population [23], we found no significant association between MAFLD and CVE in our cohort.

Finally, there may be additional factors that have an impact on the onset of metabolic comorbidities and cardiovascular risk. Our study did not reveal any clear association between the type of immunosuppressive therapy and its possible role. Even those patients who were taking everolimus (a non-CNI that may increase the risk of hyperlipidemia) did not exhibit a higher risk of cardiovascular events.

The strengths of the present study are the rather large sample size, the long median follow-up (118.5 months), and the possibility of direct access to patient information.

The limitations of this study are related to its observational and retrospective nature. Some data were missing, as shown in Tables 1 and 4. We cannot completely exclude the possibility that patients' reporting of some CVEs may have been incomplete.

Additionally, important risk factors (e.g., cigarette smoking or a family history of CVEs) have not been considered, as this information is not always available for each patient.

From a clinical point of view, the present results suggest that special attention should be given to preexisting diabetes, as these patients are at increased risk of cardiovascular morbidity. Patients with preexisting DM could benefit from tighter treatment of diabetes and, at the same time, a strict control of other risk factors for cardiovascular disease, such as hyperlipidemia, arterial hypertension, and overweight. A multidisciplinary approach involving a cardiologist and a diabetic center is recommended.

Patients with de novo metabolic syndrome have an increased risk of CVe; thus, even if de novo DM as a single risk factor is not significantly correlated with CV, it deserves attention in the context of de novo metabolic syndrome.

## 5. Conclusions

Future prospective studies on large cohorts of liver transplant recipients are needed to better stratify cardiovascular risk after LT and how to act on it: what are the therapeutic interventions and whether they should differ from those applied in the general population. This becomes even more relevant considering the increasing survival of liver transplant patients, the increasing age at transplantation, and the increasing frequency of metabolic etiology of liver disease. For all of these reasons, it is necessary to continue research in this area to identify the best strategy for follow-up and therapy of these patients.

## Figures and Tables

**Table 1:** Characteristics of the overall cohort and in patients with and without cardiovascular events (CVEs).

	Entire cohort	CVE	No CVE
	(*n* = 356)	(*n* = 63)	(*n* = 293)
Males, *n* (%)	277 (77.8)	59 (94)	218 (78.7)
Age (years) at OLT, median (IQR)	56 (49–60)	58 (51–61)	55 (48–60)
Age (years) at last follow-up, median (IQR)	64 (57–71)	66 (62–72)	64 (55–71)
Etiology of liver disease			
Alcohol, *n* (%)	107 (30.1)	28 (44)	79 (27.0)
Viral hepatitis, *n* (%)	196 (55.1)	32 (51)	163 (55.6)
HCV	149 (41.8)	20 (31.7)	129 (44)
HBV	47 (13.2)	12 (19)	35 (11.9)
Nonalcoholic steatohepatitis (NASH), *n* (%)	20 (5.6)	4 (6)	16 (5.5)
Autoimmune/cholestatic, *n* (%)	20 (5.6)	3 (5)	17 (5.8)
Other, *n* (%)	56 (15.7)	3 (5)	53 (18.1)
Death, *n* (%)	78 (21.9)	14 (22)	64 (22.0)
BMI at transplant, median (IQR)	25 (22.6–28.1)	25.7 (23.7–29.8)	24.7 (22.4–27.7)
BMI at last follow-up, median (IQR)	25.9 (23.2–28.7)	26.6 (24.3–29.8)	25.6 (23.0–28.4)
Change in BMI (median, IQR)	0.7 (1.7; 2.5)	0.9 (2.4; 2.2)	0.5 (1.6; 2.7)
Diabetes pre-OLT* n* (%)	93 (26.1)	29 (46)	64 (21.8)
De novo diabetes *n* (%)	74 (20.8)	15 (24)	59 (20.1)
Arterial hypertension pre-OLT* n* (%)	73 (20.5)	17 (27)	56 (19.1)
De novo arterial hypertension *n* (%)	143 (41.0)	33 (52)	110 (38.5)
Hyperlipidemia pre-OLT *n* (%)	14 (3.9)	5 (8)	9 (3.1)
De novo hyperlipidemia *n* (%)	153 (43.0)	41 (65)	112 (38.2)
Metabolic syndrome pre-OLT* n* (%)^*∗*^	17/322 (5.3)	6/59 (10)	11/263 (4.2)
De novo metabolic syndrome *n* (%)^*∗*^	34/322 (10.6)	12/59 (20)	22/263 (8.4)
MAFLD pre-OLT* n* (%)^*∗*^	20/256 (7.8)	3/45 (7)	17/211 (8.1)
De novo MAFLD *n* (%)^*∗*^	57/256 (22.3)	11/45 (24)	46/211 (21.8)

(^*∗*^) calculated for the subgroup of patients with complete information.

**Table 2:** Prevalence of metabolic alterations before and after liver transplantation.

	Absent pre-OLT and post-OLT	Present pre-OLT and absent post-OLT	Present pre-OLT and present post-OLT	Absent pre-OLT and present post-OLT(de novo)
		(Prevalent cases)		(Incident cases)
Diabetes mellitus type 2 *N* (%)	189 (53.1)	8 (2.2)	85 (23.9)	74 (20.8) AR^*∗*^ = 74/263 = 28.1%
Hypertension *N* (%)	140 (39.3)	6 (1.7)	67 (18.8)	143 (40.2) AR^*∗*^ = 143/283 = 50.5%
Dyslipidemia *N* (%)	189 (53.1)	3 (0.8)	11 (3.1)	153 (43) AR^*∗*^ = 153/342 = 44.7%
MAFLD *N* (%)	179 (50.3)	10 (2.8)	10 (2.8)	57 (16) AR^*∗*^ = 57/236 = 24.2%
Metabolic syndrome *N* (%)	271 (84.2%)	6 (1.9)	11 (3.4)	34 (10.6) AR^*∗*^ = 34/305 = 11.1%

^*∗*^AR, absolute risk: this measure was computed for incident cases, thus excluding prevalent cases at baseline.

**Table 3:** Incidence rates of CVEs among all transplanted patients.

	Number of patients (%)	Percentage of CVE (%)
Transient ischemic attack (TIA)	2 (0.6)	3
Acute myocardial infarction (MI)	16 (4.5)	25
Peripheral vasculopathy	13 (3.7)	21
Stroke	17 (4.8)	27
Heart failure	8 (2.2)	13
Cardiac arrest	1 (0.3)	2
Non-MI ischemic heart disease	6 (1.7)	10

**Table 4:** Simple and multiple Cox regression analyses with cardiovascular events as the primary endpoint. Fine-gray competing risk regression analysis with cardiovascular event as the primary endpoint and CVE-unrelated mortality as the competing event.

Factors	Contrast	Cox regression						Fine-gray competing risk regression		
		Univariable			Multivariable			Multivariable		
		HR	95% CI	*p*values	HR	95% CI	*p*value	SHR	95% CI	*p*values
Sex	*F* vs. *M*	0.23	0.08–0.63	0.004	0.34	0.12–0.94	0.038	0.25	0.09–0.69	0.008

Age at OLT	10-year increase	1.51	1.13–2.01	0.006	1.43	1.02–2.02	0.040	1.44	1.05–1.97	0.022

Etiology alcohol	vs. all others	2.33	1.41–3.86	<0.001	1.31	0.70–2.46	0.400			

Etiology viral hepatitis	vs. all others	0.73	0.44–1.20	0.215						

BMI at OLT	1 kg/m^2^ increase	1.08	1.01–1.15	0.019	1.05	0.97–1.12	0.236			

Diabetes	Pre-OLT vs. absent	4.62	2.55–8.38	<0.001	3.10	1.60–6.03	0.001	2.90	1.62–5.18	<0.001
	De novo vs. absent	1.77	0.90–3.50	0.098	1.27	0.59–2.71	0.538	1.37	0.69–2.72	0.370

Hypertension	Pre-OLT vs. absent	3.05	1.48–6.30	0.003	1.95	0.83–4.62	0.128			
	De novo vs. absent	1.97	1.04–3.76	0.039	1.14	0.55–2.37	0.723			

Dyslipidemia	Pre-OLT vs. absent	4.30	1.58-11.67	0.004	4.68	1.60–13.65	0.005	4.23	1.56–11.48	0.005
	De novo vs. absent	2.93	1.66–5.16	<0.001	2.21	1.17–4.16	0.014	2.35	1.32–4.18	0.004

MAFLD (missing values = 100)	Pre-OLT vs. absent	0.81	0.25–2.65	0.724						
	De novo vs. absent	0.99	0.50–1.98	0.991						

Metabolic syndrome (missing values = 34)	Pre-OLT vs. absent	3.24	1.36–7.68	0.008	3.13	0.90-10.87	0.072			
	De novo vs. absent	2.23	1.17–4.24	0.015	1.19	0.51–2.38	0.811			

Therapy	NonCNI vs. CNI	1.67	0.87–3.19	0.122						
	CNI + NonCNI vs. CNI	1.01	0.57–1.79	0.965						

## Data Availability

The retrospective data used to support the findings of this study may be released upon application to the corresponding author prof. Manuela Merli, who can be contacted at manuela.merli@uniroma1.it.
